# The Fragility of Statistical Significance in the Use of Aspirin in Prevention of Venous Thromboembolism Events Following Total Joint Arthroplasty: Systematic Review and Meta-Analysis of Randomized Controlled Trials

**DOI:** 10.3390/jcm13216369

**Published:** 2024-10-24

**Authors:** Tyler K. Williamson, Victor H. Martinez, Luke Verlinsky, Jacob L. Brennan, Frank A. Buttacavoli

**Affiliations:** Department of Orthopaedic Surgery, University of Texas Health San Antonio, San Antonio, TX 78229, USA; vic.martinezz43@gmail.com (V.H.M.); verlinsky@uthscsa.edu (L.V.);

**Keywords:** total joint arthroplasty, chemoprophylaxis, hip arthroplasty, knee arthroplasty, deep vein thrombosis, venous thromboembolism, prophylaxis, aspirin

## Abstract

**Background/Objectives:** Comparative studies often use the *p* value to convey statistical significance, but fragility indices (FI) and fragility quotients (FQ) may better signify statistical strength. The use of aspirin as venous thromboembolism (VTE) chemoprophylaxis following elective arthroplasty has been debated between the orthopedic and cardiac fields. The purpose of this study was to apply both the FI and FQ to evaluate the degree of statistical fragility in the total joint arthroplasty (TJA) literature regarding aspirin (ASA) use for VTE prevention. **Methods:** We performed a systematic search for TJA clinical trials from 2004 to 2023 reporting comparisons between ASA and other chemoprophylaxis methods for VTE. The FI of each outcome was calculated through reversal of a single outcome event until significance was reversed. The FQ was calculated by dividing each fragility index by study sample size and interquartile range (IQR) was calculated. SPSS Meta-analysis function was used to calculate the Mean Effect Size Estimate and 95% Confidence Intervals for each outcome. **Results:** Of 245 articles screened, 39 met search criteria, with 10 RCTs included for analysis (n = 11,481 patients). There were 38 outcome events reported, with three significant (*p* < 0.05) outcomes and 35 non-significant (*p* > 0.05) outcomes identified. The overall FI and FQ for all 38 outcomes were 6 (IQR: 5–7) and 0.059 (IQR: 0.044–0.064), respectively. Seven studies (70%) reported a loss-to-follow-up (LTF) greater than the overall FI. There was no increased risk of DVT, PE, or mortality with use of ASA (all *p* > 0.2). **Conclusions:** Despite showing non-inferiority in preventing venous thromboembolic events in TJA overall, the highest-level peer-reviewed literature concerning aspirin use following total joint arthroplasty is considered statistically fragile due to high loss-to-follow-up. In addition to the reporting of the *p* value, the fragility index and quotient can further provide insight into the strength and trustworthiness of outcome measures.

## 1. Introduction

Total joint arthroplasty (TJA), comprising both total hip (THA) and total knee arthroplasty (TKA), consistently produces favorable outcomes in 85–100% of patients, but postoperative complications, particularly venous thromboembolism (VTE), remain a significant and prevalent concern and can lead to substantial morbidity, increased healthcare costs, and mortality [1,2,3,4,5]. Traditionally, pharmacological agents such as low-molecular-weight heparin (LMWH) and rivaroxaban have been mainstay prophylactic treatment, but these come with their own limitations, including the risk of bleeding, increased cost, patient compliance, and the need for routine monitoring [6,7]. Recent studies have explored the potential of aspirin (ASA) as an alternative prophylactic strategy for VTE following TJA, considering its established safety profile, cost effectiveness, and ease of administration [8].

Numerous clinical trials and observational studies have examined the efficacy and safety of aspirin as a prophylactic measure against VTE after TJA [8,9,10,11,12,13,14,15,16]. Notably, an appropriately powered clinical trial out of the Hospital for Special Surgery found no difference between LMWH and ASA after TKA [13]. Yet a multicenter trial by the CRISTAL study group found aspirin to be inferior to LMWH in prevention of VTE following TJA [8]. More recently, the International Consensus Meeting on Venous Thromboembolism (ICM-VTE) agreed in majority that aspirin is currently the safest and most effective method of chemoprophylaxis following TJA in light of current evidence [17]. These recommendations were further iterated through a survey of the American Board of Orthopaedic Surgery (ABOS) Part II candidates who most often used aspirin as chemoprophylaxis and demonstrated the lowest rates of complications in doing so [18]. A national database study validated these findings, highlighting an increase in aspirin regimens by 800% postoperatively from 2012 to 2022 in both low- and high-risk patients [19]. In addition to this increase in prescription, low-dose aspirin was also found to be associated with similar or lower rates of all relevant complications, including DVTs and PEs. However, recent reviews and commentaries caution against its use due to limited and low-quality evidence available on this topic [20,21]. The conflicting findings across these investigations raise questions about the statistical robustness and fragility of the reported effects. And though aspirin may ultimately be as effective in VTE prevention, it is essential to assess the stability and reliability of the reported findings.

The concept of statistical fragility investigates the susceptibility of study results to small changes in methodology, sample size, effect size, and even loss-to-follow-up. Statistical fragility refers to the number of events that would have needed to change in order to change the significance (or non-significance) of a certain comparison. For instance, a study may show aspirin to be as effective as a comparator in an effort to prevent VTE occurrence (5% vs. 3%, *p* > 0.05). However, if the study has a small sample size (50 received aspirin and 50 received the comparator), only a relatively small number of events may be needed to change the non-significant outcome to a significant difference (i.e., if two aspirin patients developed a DVT, the overall difference would now be 7% vs. 3% and the difference would become significant [*p* < 0.05]). Given the impact the choice of chemoprophylactic agent has on a patient’s postoperative course, understanding the strength of evidence regarding the use of aspirin is crucial in patient care to properly weigh financial and clinical efficacy.

In this systematic review, we aim to critically evaluate the existing literature on aspirin for VTE chemoprophylaxis following TJA, by answering two questions: “What is the statistical strength of current literature examining aspirin as chemoprophylaxis following TJA?” and “Is aspirin non-inferior in regards to VTE and mortality prevention following TJA?”. By synthesizing the available evidence, we seek to determine the overall effect of aspirin as a prophylactic measure and assess the robustness of the observed results to provide valuable insights for clinical practice and contribute to the optimization of postoperative care of patients following TJA.

## 2. Materials and Methods

### 2.1. Systematic Review Registration

This systematic review followed the Preferred Reporting Items for Systematic reviews and Meta-Analyses (PRISMA) guidelines and was exempt from institutional review board (IRB) approval as it utilized publicly accessible data (Appendix A) [22]. The review protocol was registered with International Prospective Register of Systematic Reviews (PROSPERO) before initiating data collection (registration ID: CRD42024511992).

### 2.2. Study Design and Eligibility Criteria

We conducted searches in the PubMed, Cochrane, and SCOPUS databases from 2004 to 2023 for prospective randomized controlled trials (RCTs) that investigated the use of aspirin for preventing venous thromboembolism (VTE) after elective total joint arthroplasty (TJA). The search terms included “aspirin arthroplasty trial”, “aspirin arthroplasty”, and “thromboembolism trial arthroplasty”. To be included, studies had to be randomized, prospective in nature, and provide dichotomous outcomes for VTE complications along with associated *p* values in order to properly calculate statistical fragility [23]. Exclusion criteria included retrospective studies, systematic reviews, meta-analyses, animal research, crossover trials, studies with mixed interventions, those focused on arthroplasty after femoral fractures, and studies lacking dichotomous outcomes. Studies solely focused on traumatic indications for total hip arthroplasty or those unable to differentiate between elective and traumatic cases were also excluded as these entities have differing risks of complications and would create further heterogeneity in our desired population [24,25].

### 2.3. Identification of Studies

After the initial search, we screened titles for relevance to aspirin compared with other VTE chemoprophylaxis options, followed by a detailed examination of the methods and results in the remaining studies. Abstracts were also reviewed to confirm that studies involved human subjects and were published in English.

### 2.4. Article Review Process and Data Collection

Two authors (V.M. and T.K.W.) independently reviewed the abstracts before conducting full-text assessments to evaluate methodologies and the criteria for inclusion and exclusion. Studies that compared multiple chemoprophylactic agents were analyzed as distinct comparisons if there was no overlap among the groups. Data were extracted into a spreadsheet, organizing variables accordingly.

### 2.5. Methodological Quality and Bias Assessment

We used the Risk of Bias (RoB) Version 2.0 tool developed by Cochrane to assess methodological biases in the included studies. Two independent reviewers (T.K.W. and V.H.M.) evaluated all studies, resolving discrepancies through consensus with a third reviewer (L.V.) [26].

The GRADE approach was employed to evaluate the certainty of evidence, with two authors (T.K.W. and V.H.M.) downgrading evidence based on established principles [9,13]. GRADE assessments were completed for each individual variable undergoing meta-analysis.

### 2.6. Study Characteristics

We collected various details from each study meeting the inclusion criteria, including title, publication year, study location, patient sample size, type of joint involved (hip or knee), number of patients lost to follow-up, demographic information, study outcomes (DVT, PE, readmission, mortality), VTE chemoprophylaxis regimen, dosage, duration, and reported *p* values. We constructed 2 × 2 contingency tables for each dichotomous outcome.

### 2.7. Calculation of Fragility Index and Fragility Quotient

Using established methods, the Fragility Index (FI) was calculated for all categorical, dichotomous outcomes reported in the studies [23]. We recalculated *p* values using Fisher’s exact test, adjusting event counts stepwise until the *p* values indicated statistical significance or insignificance, determining either FI or reverse FI (RFI). The corresponding Fragility Quotient (FQ) was then calculated by dividing the FI by the sample size. Previous studies and the American Academy of Orthopaedic Surgeons (AAOS) have determined a mean FI of 2 or greater to demonstrate relative statistical strength, which was used as a threshold to guide our study [27]. Loss-to-follow-up (LTF) was also measured and statistical strength was negated if the overall FI of the outcome was less than the study LTF.

### 2.8. Statistical Analysis

Descriptive statistics were calculated for each study and outcome. Primary outcomes included DVT rates, while secondary outcomes encompassed rates of PE, major bleeding events, readmissions, and mortality. We computed frequency distributions and summary statistics for all variables, employing chi-squared and *t*-tests for group comparisons.

Meta-analysis was performed when a sufficient number of studies provided data for an outcome, assessing heterogeneity using the Cochrane Q and I^2^ statistics [23]. We calculated 95% confidence intervals (CIs) to evaluate heterogeneity when applicable. Sensitivity analyses were conducted to identify outliers and perform influence analysis, alongside standard meta-analytic summaries [22,23,26,28]. Given the high variability between variable recording in studies, not all relevant variables were able to be accounted for in meta-regression to assess for effects on the relationship between ASA and outcomes. However, age and sex were commonly recorded and were included in the meta-regression component of the SPSS function.

Mean effect size estimates (MESE) were derived from final means, standard deviations, and sample sizes for both intervention and control groups, with negative values indicating a protective effect for the intervention group. Sub-analyses focused on total-hip-arthroplasty (THA) and total-knee-arthroplasty (TKA) populations. Data analysis was completed in February 2024 using SPSS software (IBM SPSS Statistics for Windows, v29.1.1). Statistical significance was set at *p* < 0.05.

## 3. Results

### 3.1. Study Inclusion

**Figure 1:**
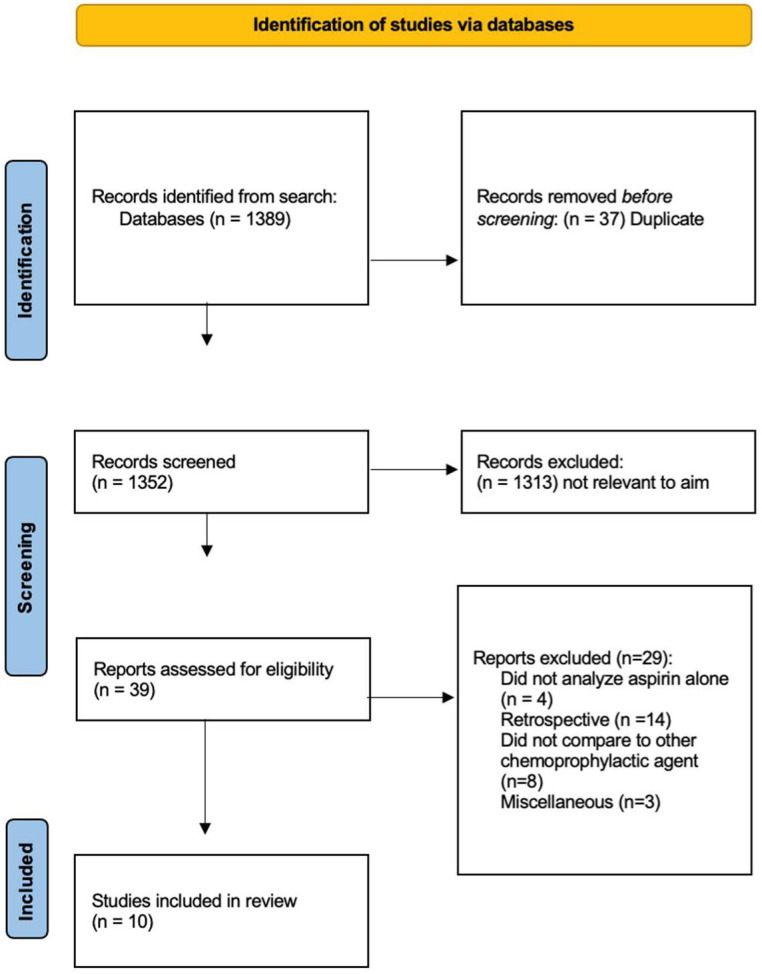
PRISMA flowchart.

Of the 1389 studies screened, 39 met the search criteria with 10 RCTs included in the analysis (Figure 1, Table A1). Amongst the comparisons included, 11,371 unique patients underwent elective joint arthroplasty and were enrolled. The follow-up data to meet inclusion within the studies were present for 10,801 patients (95.0%). Regarding intervention, 6224 patients (57.6%) were prescribed aspirin for VTE chemoprophylaxis following surgery (Table 1). Thirty-eight events with three significant (*p* < 0.05) outcomes and 35 with non-significant (*p* > 0.05) outcomes were identified. In total, 314 patients (2.9%) developed a DVT within the postoperative period, while 119 patients (1.1%) developed either a PE or MBE and eight patients expired (0.1%; Table 2).

### 3.2. Methodological Bias of Studies

The risk of bias of included studies was deemed overall acceptable, with a low risk of bias observed in the majority of domains (Table A2). The interobserver reliability of bias assessment was excellent (ICC: 0.93, 95% confidence interval (CI): 0.88 to 0.95). Evidence quality was assessed with the GRADE rubric and recorded in Table A3. Notably, all three variables undergoing meta-analyses were judged as medium quality.

### 3.3. Overall Outcome Analysis

For the three outcomes that were reported as significant, the median number of events required to change significance was 5 (IQR: 5–25) and the median FQ was 0.024 (IQR: 0.010–0.030; Table 3). For the 35 non-significant outcomes, the median number of events required to change significance was 6 (IQR: 5–7) and the median FQ was 0.130 (IQR 0.088–0.180). Of the 38 total outcomes, 10 (26.3%) were primary and 28 (73.7%) were secondary. For the outcomes where FI < LTF (n = 26), the median FI was found to be 3.5 (IQR 3–5.5). For the outcomes where FI > LTF (n = 12), the median FI was found to be 5 (IQR 4–6).

### 3.4. Primary and Secondary Outcomes

The median FI for primary outcomes was 8 (IQR 5.5–11.5; Table 4) and the median FQ was 0.090, indicating that the reversal of 9 of 100 outcomes may change the study significance of the included studies. Of the ten primary outcomes recorded, five outcomes reported loss-to-follow-up (LTF) data greater than the overall median FI of 6. Therefore, 50.0% of studies reported an LTF value that was greater than the overall FI. The median FQ for secondary outcomes was 0.039, indicating that reversal of 4 of 100 outcomes may change study significance (Table 4). Twenty-one secondary outcomes reported LTF data greater than 6 (75.0%).

### 3.5. Chronological Analysis

Fragility sub-analysis per year of publication identified a median FI of 6 (IQR: 5–8) and 61.9% of less-than-robust outcomes from 2004 to 2014. A median FI of 5 (IQR: 5–8) and 76.5% of less-than-robust outcomes was found from 2015 to 2023, thus demonstrating that the strength of statistical significance concerning this topic over the 20-year period has not improved.

### 3.6. Meta-Analysis: Deep Vein Thrombosis

**Figure 2:**
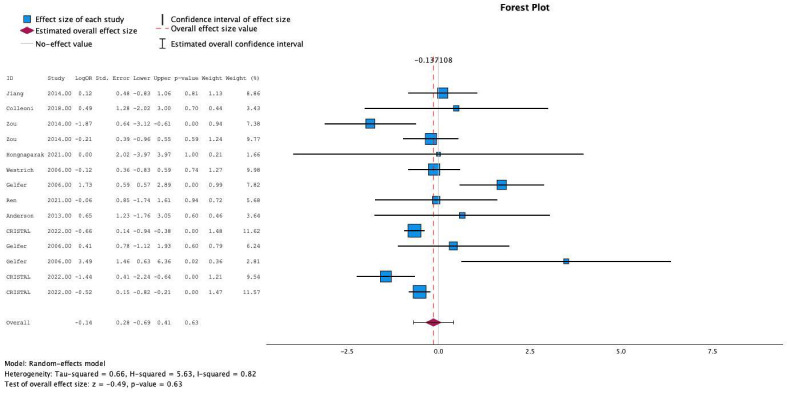
Effects of aspirin on development of deep vein thrombosis [8,9,10,11,12,13,14,15,16].

All ten studies individually reported the relationship between postoperative anticoagulation and the development of DVT. The test for heterogeneity was significant and the studies had poor heterogeneity (*I*^2^ = 72%). In these studies, the use of aspirin was not more likely to be associated with development of DVT compared to other prophylactic agents (Mean Effect Size Estimate (MESE) = −0.1, 95% CI: [−0.7–(0.5)]; Z = −0.3; *p* = 0.8 [Figure 2]). This was not affected by testing in meta-regression with continuous age and sex.

### 3.7. Meta-Analysis: Pulmonary Embolism

**Figure 3:**
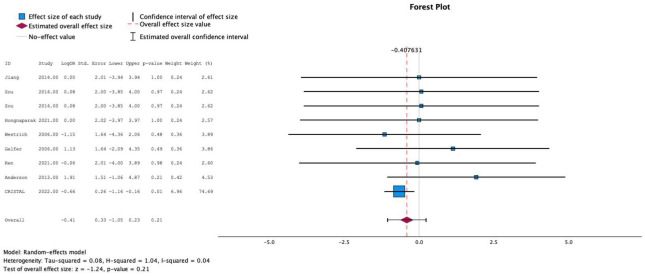
Effects of aspirin on development of pulmonary embolism [8,9,11,12,13,14,15,16].

Nine studies individually reported the relationship between postoperative anticoagulation and the development of PE. The test for heterogeneity was significant and the studies had moderate heterogeneity (*I*^2^ = 4%). In these studies, the use of aspirin was not more likely to be associated with development of PE compared to other prophylactic agents (MESE = −0.4, 95% CI: [−1.1–(0.2)]; Z = −1.2; *p* = 0.2 [Figure 3]). This was not affected by testing in meta-regression with continuous age and sex.

### 3.8. Meta-Analysis: Mortality

**Figure 4:**
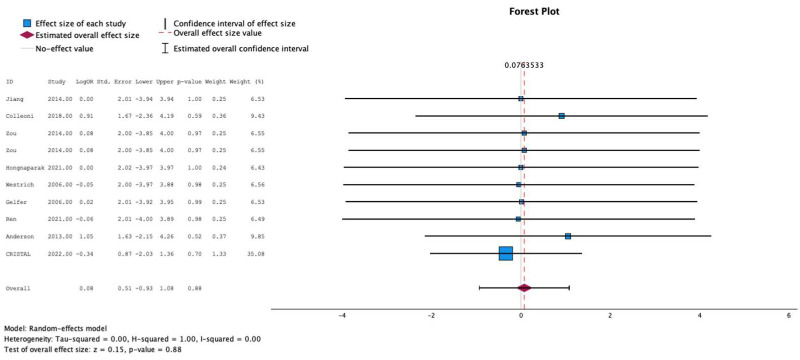
Effects of aspirin on mortality [8,9,10,11,12,13,14,15,16].

All ten studies individually reported the relationship between postoperative anticoagulation and mortality. The test for heterogeneity was significant and the studies had moderate heterogeneity (*I*^2^ = 0%). In these studies, the use of aspirin was not associated with increased mortality compared to other prophylactic agents (MESE = 0.1, 95% CI: [−0.9–(1.1)]; Z = 0.2; *p* = 0.9 [Figure 4]). This was not affected by testing in meta-regression with continuous age and sex.

### 3.9. Meta-Analysis: Sub-Analysis of Effects Within THA vs. TKA

**Figure 5:**
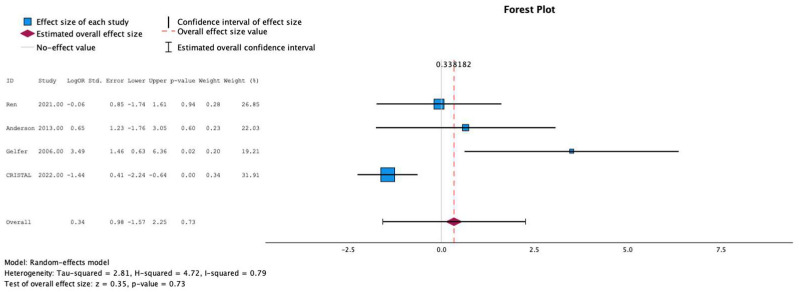
Effects of aspirin on development of deep vein thrombosis in THA [14,15,16].

**Figure 6:**
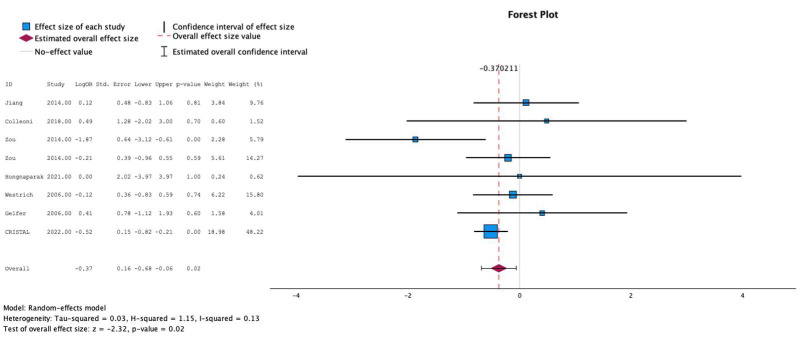
Effects of aspirin on development of deep vein thrombosis in TKA [8,9,10,11,12,13,14].

Four studies individually reported the relationship between postoperative anticoagulation and development of DVT in THA. In these studies, the use of aspirin was not associated with increased risk of DVT (MESE = 0.3, 95% CI: [−1.6–(2.3)]; Z = 0.4; *p* = 0.7 [Figure 5]). Eight studies individually reported the relationship between postoperative anticoagulation and development of DVT in TKA. In these studies, the use of aspirin was associated with increased risk of DVT compared to other agents overall, especially LMWH (MESE = 0.4, 95% CI: [0.1–0.7]; Z = 2.3; *p* = 0.02 [Figure 6]).

## 4. Discussion

This study aimed to evaluate the strength of clinical trials regarding the role of aspirin in VTE prophylaxis following TJA by utilizing an FI and FQ analysis. Our study reported an overall FI and FQ for all 38 outcomes of 6 (IQR: 5–7) and 0.059 (IQR: 0.044–0.064), respectively. Furthermore, seven studies (70%) reported a loss-to-follow-up (LTF) greater than the overall FI, suggesting that most of the highest-level peer-reviewed literature concerning aspirin use following TJA is less than robust. These findings further emphasize the recent push in orthopedic literature for future statistical reporting to include FI and FQ in conjunction with *p* values to provide a comprehensive data evaluation [28,29,30].

As VTE prophylaxis following TJA continues to be a highly studied yet controversial topic, a recent analysis by Boucher et al. assessed the fragility of 32 RCTs evaluating VTE prophylaxis following TJA [28]. Their findings reported an FI of 7 and an FQ of 0.01, with 42.3% of outcomes having an LTF greater than the FI, suggesting that current evidence lacks statistical stability, but did not report the fragility of individual prophylactic medications, such as aspirin. With previous systematic reviews demonstrating varying results amongst VTE prophylaxis agents following TJA, there is a need to assess the outcomes of individual interventions to provide more detailed recommendations [6]. Furthermore, there have been two recent major RCTs reporting on outcomes regarding VTE development with the use of aspirin prophylaxis in comparison to enoxaparin and LMWH following lower extremity joint arthroplasty and fractures with conflicting results, therefore stressing the importance of further research on this topic [8,31]. Our study provides further awareness regarding statistically fragile evidence involving aspirin use for VTE prophylaxis following TJA.

VTE complications are severe and contribute to significantly diminished patient outcomes, with increased pain, length of stay, and notably hemodynamic instability recorded in 11% of patients who sustain acute in-hospital PE after total knee arthroplasty [32]. Patients who sustain inpatient VTE complications are more likely to develop associated complications such as pneumonia, urinary tract infections, sepsis, myocardial infection, and stroke as well [32]. VTE events have also been associated with an approximately $20,000 increase in hospital-associated charges after primary joint arthroplasty, and up to $50,000 in the revision setting [33]. Further research is paramount in preventing the increased burden of VTE complications on both patients and the global healthcare system.

Recently, the American Academy of Orthopaedic Surgeons (AAOS) issued clinical practice guidelines for evaluating research, with a median FI of 2 deemed to constitute “strong evidence” [27]. The number of events required to change significance in our study was 6, suggesting relative statistical strength. However, these data should be interpreted cautiously, as 70% of studies demonstrated loss-to-follow-up (LTF) data greater than the overall FI, suggesting that better maintenance of follow-up may have reversed the conclusions [34]. While previous orthopedic studies have also highlighted the prevalence of statistically fragile results, no study has reported a higher LTF than the outcomes of our study [28,35]. While caution is advised when interpreting the data on aspirin usage in VTE prophylaxis following TJA, this reveals the significant opportunity to supplement *p* values with a fragility index and quotient in critical trials such as those included in this review to provide further insight into the strength and trustworthiness of outcome measures in this complex topic. Surgeon researchers should be wary of the possible bias encroaching on the findings of trials due to loss-to-follow-up and the impact this may have on the strength of their recommendations. Therefore, thorough dedication and appropriate allocation of resources should be accounted for to retain patients within the protocol of the study to best ensure the validity of their findings.

A potential area of conflict in patient care currently lies within combined orthopedic and hospitalist postoperative patient management after TJA. Prior to 2012, the American College of Chest Physicians guidelines were in opposition to the American Academy of Orthopaedic Surgeons guidelines with regards to treatment with aspirin in the perioperative period [36]. The 2019 update to the National Institute for Health and Care Excellence (NICE) guidelines out of the United Kingdom recommends 10 days of LMWH treatment followed by 28 days of aspirin therapy in elective hip replacement [37]. With increased use of a multidisciplinary approach and co-management of postoperative patients with the use of hospitalist services, it is paramount that orthopedic surgeons and medicine physicians have a strong consensus in the management of TJA patients [38]. Our data support the conclusion that ASA is indeed non-inferior to other chemoprophylactic agents overall in THA, but may have less of a protective effect in TKA patients, aligning with the results found in the CRISTAL trial and a recent secondary analysis [8,39]. To make co-management recommendations, orthopedic surgeons must have a thorough understanding of both the strengths and limitations of the available evidence. Additionally, this study emphasizes that risk stratification and patient profiling are crucial; aspirin may not be the agent of choice in every patient, and a thorough patient evaluation should guide management practices. These findings ultimately suggest there is room to conduct higher-quality studies with high rates of follow-up to further boost the results found in the included trials. In addition, surgeons and physicians should utilize these findings in conjunction with their own clinical experience to critically evaluate the best form of chemoprophylaxis for each patient.

### Limitations

While we believe a fragility analysis should be included in comparative trials, our findings are not without limitations. First, the research is limited as only ten RCTs were eligible for analysis. The FI and FQ can only be applied to dichotomous outcomes, excluding the analysis of continuous variables. Both of these limit the number of studies that could be included within our review as well as other relevant continuous variables that may have been helpful (length of stay, patient-reported outcomes, etc.). However, inclusion of only RCTs increases the mean certainty and quality of evidence present within the study, strengthening the findings and recommendations overall. Multiple chemoprophylactic agents were recorded in comparison to aspirin and this may limit the direct generalizability of these findings to clinical decisions made by physicians on a case-by-case basis. Moreover, FI does not consider the sample size of the study. FQ was developed to mitigate some of these limitations, but even FQ is not constrained by an actual fragility threshold or lack thereof. Unlike the *p* value, there is no threshold for interpreting the significance of reported fragility values. Lastly, fragility analysis is only one component of a comprehensive evaluation of the robustness of a study, and it should not be interpreted independently of the *p* value.

## 5. Conclusions

Despite showing non-inferiority in preventing venous thromboembolic events in TJA overall and within the THA cohort, the majority of highest-level peer-reviewed literature concerning aspirin use following total joint arthroplasty is less than robust, with more than two-thirds of studies considered statistically fragile. In addition to the reporting of the *p* value, the fragility index and quotient can further provide insight into the strength and trustworthiness of outcome measures.

## Figures and Tables

**Table 1:** List and proportion of chemoprophylactic agents in analysis.

Type of Intervention	Number of Patients	Proportion of Total
Aspirin	6224	57.6%
Rivaroxaban	186	1.7%
LMWH	4391	40.7%

**Table 3:** Overall outcomes.

Type of Outcome	Value	IQR
Total Fragility Index (n = 38)	6	5–8
Total Fragility Quotient (n = 38)	0.080	0.027–0.110
Fragility Index when FI > LTF (n = 12)	5	4–6
Fragility Quotient when FI > LTF (n = 12)	0.118	0.088–0.174
Fragility Index when FI < LTF (n = 26)	3.5	3–5.5
Fragility Quotient when FI < LTF (n = 26)	0.130	0.081–0.200

**Table 4:** Primary and secondary outcomes.

Primary Outcomes	Value	IQR
Fragility Index (n = 10)	8	5.5–11.5
Fragility Quotient (n = 10)	0.090	0.030–0.106
Number of Studies with FI < LTF	5	50.0%
Secondary Outcomes	Value	IQR
Fragility Index (n = 28)	5.5	5–6.5
Fragility Quotient (n = 28)	0.039	0.017–0.064
Number of Studies with FI < LTF	21	75.0%

**Table 2:** List and rate of outcomes in analysis.

Type of Outcome (# of Comparisons)	Number of Patients	Rate of Outcome
Deep Vein Thrombosis (n = 10)	314	2.9%
Pulmonary Embolism (n = 9)	84	0.8%
Major Bleeding Event (n = 6)	35	0.3%
Readmission (n = 3)	219	2.3%
Mortality (n = 10)	8	0.1%

## Data Availability

All data are available upon request.

## Appendix A

**Table A1:** List and description of articles included in analysis.

Title	Author/ Year	Journal	Study Type	Country	Setting	Patients in Study	Number of DVTs	Other Agent
Aspirin combined with mechanical measures to prevent venous thromboembolism after total knee arthroplasty: a randomized controlled trial	Jiang 2014 [9]	Chinese Medical Journal	RCT	China	Academic Center	120	21	LMWH + Rivaroxaban
Venous thromboembolism prophylaxis after total knee arthroplasty (TKA): aspirin vs. rivaroxaban	Colleoni 2018 [10]	Revista Brasileira de Ortopedia	RCT	Brazil	Academic Center	32	3	Rivaroxaban
Administering aspirin, rivaroxaban and low-molecular-weight heparin to prevent deep venous thrombosis after total knee arthroplasty	Zou 2014 [11]	Blood Coagulation and Fibrinolysis	RCT	China	Academic Center	324	35	Rivaroxaban, LMWH
Aspirin versus rivaroxaban to prevent venous thromboembolism after total knee arthroplasty: a double-blinded, randomized controlled trial	Hongnaparak 2021 [12]	Revista Brasileira de Ortopedia	RCT	Thailand	Academic Center	40	0	Rivaroxaban
VenaFlow plus Lovenox vs. VenaFlow plus aspirin for thromboembolic disease prophylaxis in total knee arthroplasty	Westrich 2006 [13]	Journal of Arthroplasty	RCT	New York	Academic Center	191	35	LMWH
Deep vein thrombosis prevention in joint arthroplasties: continuous enhanced circulation therapy vs. low molecular weight heparin	Gelfer 2005 [14]	Journal of Arthroplasty	RCT	Israel	Academic Center	121	21	LMWH
Comparable efficacy of 100 mg aspirin twice daily and rivaroxaban for venous thromboembolism prophylaxis following primary total hip arthroplasty: a randomized controlled trial	Ren 2021 [15]	Chinese Medical Journal	RCT	China	Academic Center	70	6	Rivaroxaban
Aspirin versus low-molecular-weight heparin for extended venous thromboembolism prophylaxis after total hip arthroplasty	Anderson 2013 [16]	Annals of Internal Medicine	RCT	Canada	Multiple Academic Centers	778	3	LMWH
Effect of aspirin vs. enoxaparin on symptomatic venous thromboembolism in patients undergoing hip or knee arthroplasty: the CRISTAL randomized trial	CRISTAL 2022 [8]	JAMA	RCT	Australia	Multiple Academic Centers	9,203	190	LMWH

**Table A2:** Risk of Bias (RoB-2) analysis.

Study	Randomization	Deviation	Missing Data	Outcome Measure	Result Selection	Overall
Jiang 2014 [9]	L	L	L	L	M	L
Colleoni 2018 [10]	L	L	M	M	L	L
Zou 2014 [11]	L	L	L	L	M	L
Hongnaparak 2021 [12]	L	L	L	L	L	L
Westrich 2006 [13]	L	L	M	L	M	L
Gelfer 2005 [14]	L	L	M	L	L	L
Ren 2021 [15]	L	L	M	L	L	L
Anderson 2013 [16]	M	M	M	L	M	M
CRISTAL 2022 [8]	M	L	M	M	L	M
Overall	L	L	M	L	L	L

Abbreviations: L—low risk of bias (green); M—medium risk of bias (yellow).

**Table A3:** Grading of Recommendations Assessment, Development and Evaluation (GRADE).

Risk Factors	Number of Patients {Studies}	Quality of the Evidence {GRADE}
Deep Vein Thrombosis	11,371 {10}	Moderate ⊕⊕⊕◯ (due to risk of bias, consistency of effect)
Pulmonary Embolism	11,339 {9}	Moderate ⊕⊕⊕◯ (due to risk of bias)
Mortality	11,371 {10}	Moderate ⊕⊕⊕◯ (due to risk of bias)
